# Supplementary material for: Functional trajectories during innate spinal cord repair
Source: Front Mol Neurosci. 2023 Jul 10;16:1155754. doi: 10.3389/fnmol.2023.1155754 (PMC10365889; doi:10.3389/fnmol.2023.1155754)
Supplement: Supplementary file 1 [file Data_Sheet_1.pdf]

Functional Trajectories during innate spinal cord repair

Nicholas O. Jensen, Brooke Burris, Lili Zhou, Hunter Yamada, Catrina Reyes, Zachary Pincus, and Mayssa H. Mokalled.

[1] Department of Developmental Biology

Washington University School of Medicine, St. Louis, MO, USA

[2] Center of Regenerative Medicine

Washington University School of Medicine, St. Louis, MO, USA

[4] Author for correspondence: *mmokalled@wustl.edu*

Supplementary Figures

Figure S1

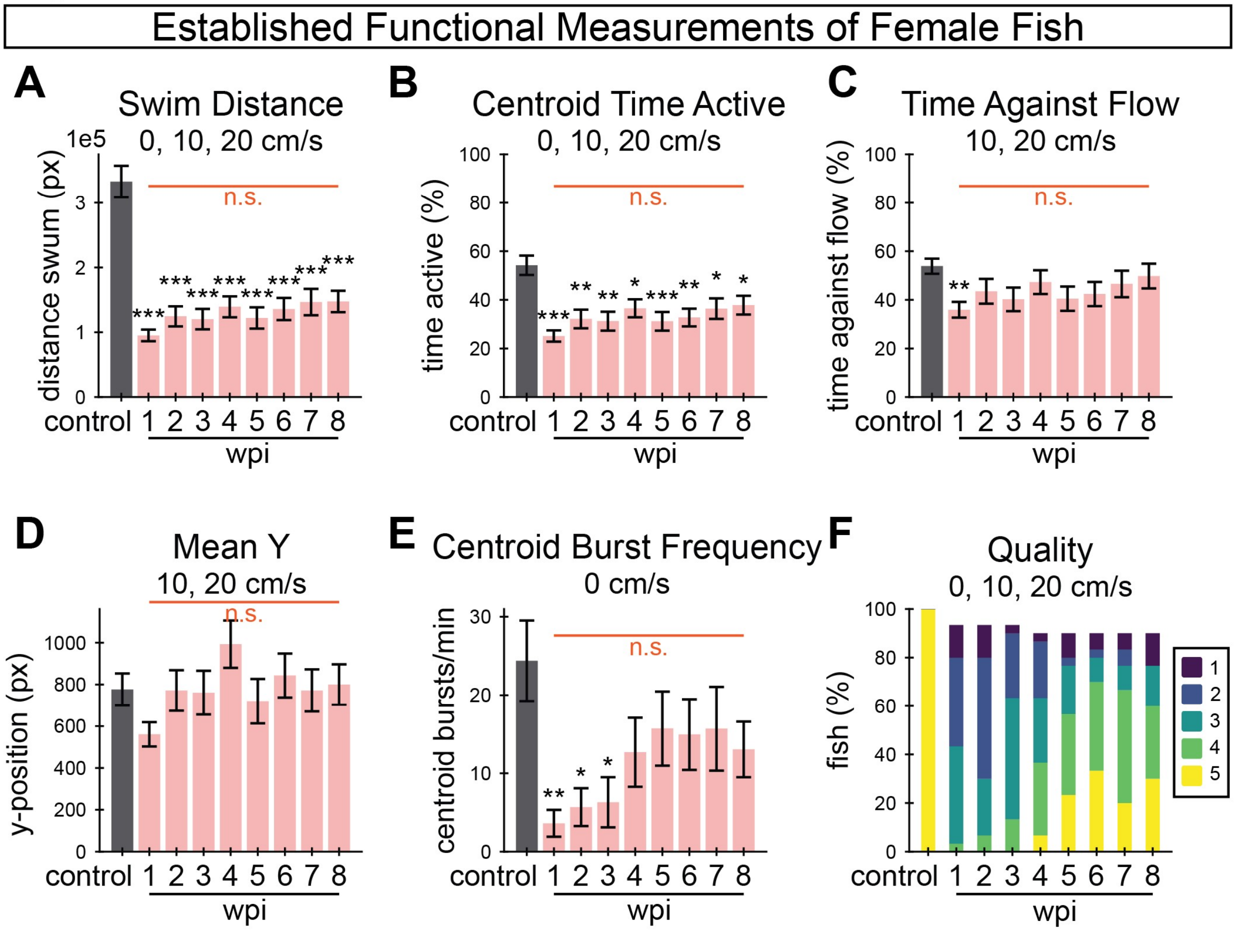

Figure S1. Established functional measurements for female fish in the tracking experiment. (A-F) A suite of established functional measurements were used to assess swim recovery for female fish following SCI. Swim distance (A), activity as measured by centroid movement (B), time swimming against the flow (C), mean y-position in the axis of flow (D), and burst frequency as measured by centroid movement (E) represent swim capacity measurements. Perceived swim quality scores are shown in F. This figure includes 30, 28, 28, 28, 27, 27, 27, 27, 27 fish at control, 1, 2, 3, 4, 5, 6, 7, 8 wpi assays, respectively. Error bars depict SEM and statistical significance was determined by Brown-Forsythe and Welch's ANOVA tests with Dunnett's T3 multiple comparisons tests. p-value markers in black represent comparisons between each time point post-injury relative to control measurements prior to injury. Red horizontal bars and p-values marked in red show significance between 1 wpi and 8 wpi. ***$P<0.001$; **$P<0.01$; *$P<0.05$; ns, $P>0.05$.

Figure S2

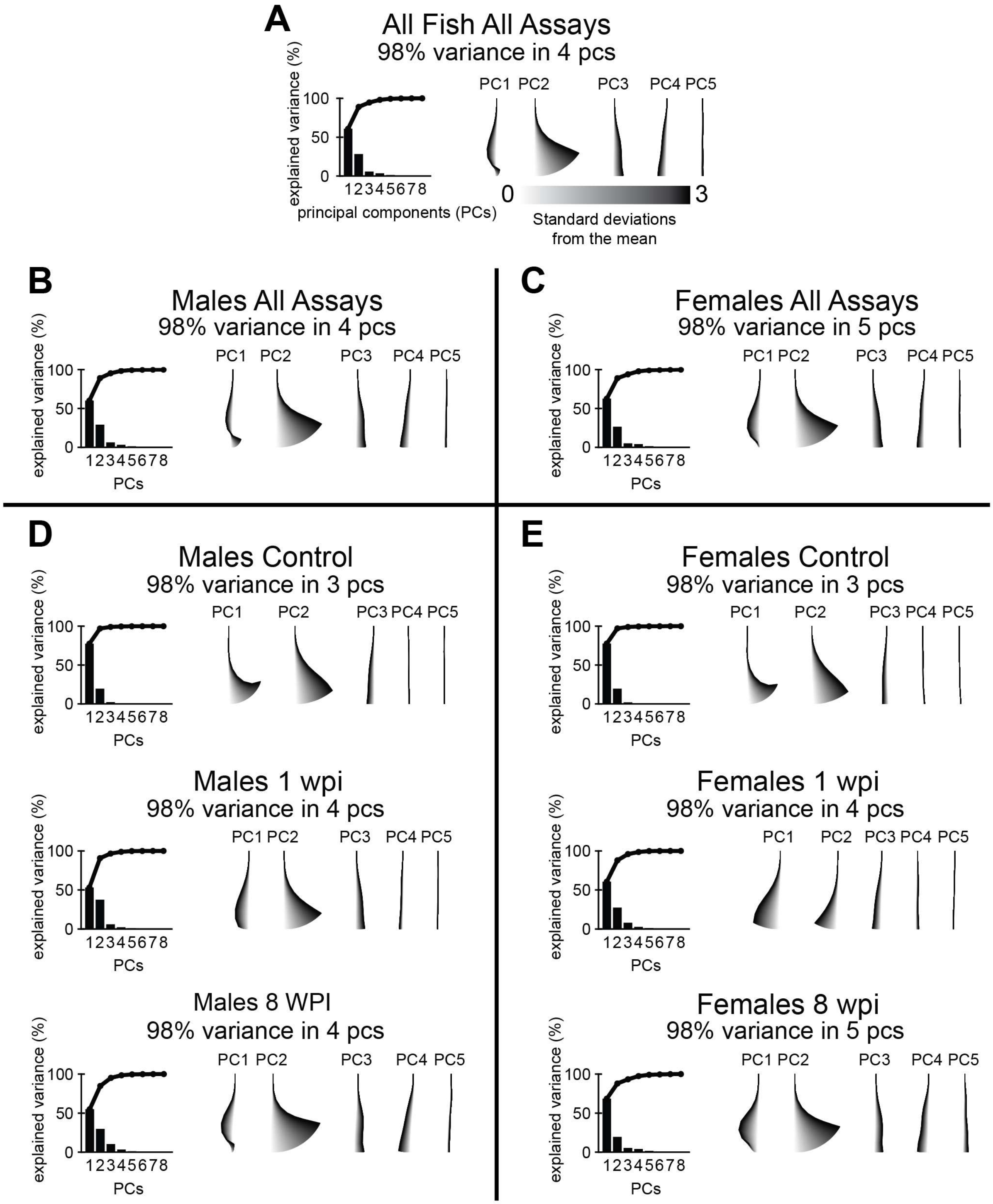
A
All Fish All Assays
98% variance in 4 pcs
explained variance (%)
100
50
0
12345678
principal components (PCs)
PC1 PC2 PC3 PC4 PC5
0
3
Standard deviations from the mean
B
Males All Assays
98% variance in 4 pcs
C
Females All Assays
98% variance in 5 pcs
D
Males Control
98% variance in 3 pcs
E
Females Control
98% variance in 3 pcs
Males 1 wpi
98% variance in 4 pcs
Females 1 wpi
98% variance in 4 pcs
Males 8 WPI
98% variance in 4 pcs
Females 8 wpi
98% variance in 5 pcs
PCs

Figure S2. Principal component analysis of angle poses observed in the tracking experiment. On the left side of each PCA is a scree plot showing the percentage of variance captured by each PC. On the right side of each PCA are the first five components plotted as “eigenfish” by adding multiples of each PC to the mean pose. **(A)** A general representation of the posture space, and its complexity, for both healthy and injured fish. PCA of poses from all fish, all assays. **(B)** PCA of poses from all assays, male fish only. **(C)** PCA of poses from all assays, female fish only. **(D)** Posture space for male fish using poses taken from control, 1 wpi, and 8 wpi assays (top to bottom). **(E)** Posture space for female fish using poses taken from control, 1 wpi, and 8 wpi assays (top to bottom). Posture space is similarly complex whether separated by sex or taken altogether. Panel A includes 60, 58, 58, 58, 56, 56, 55, 54, 54 fish at control, 1, 2, 3, 4, 5, 6, 7, 8 wpi assays, respectively. Panels B and D include 30, 30, 30, 30, 29, 29, 28, 27, 27 fish at control, 1, 2, 3, 4, 5, 6, 7, 8 wpi assays, respectively. Panels C and E include 30, 28, 28, 28, 27, 27, 27, 27, 27 fish at control, 1, 2, 3, 4, 5, 6, 7, 8 wpi assays, respectively.

Figure S3

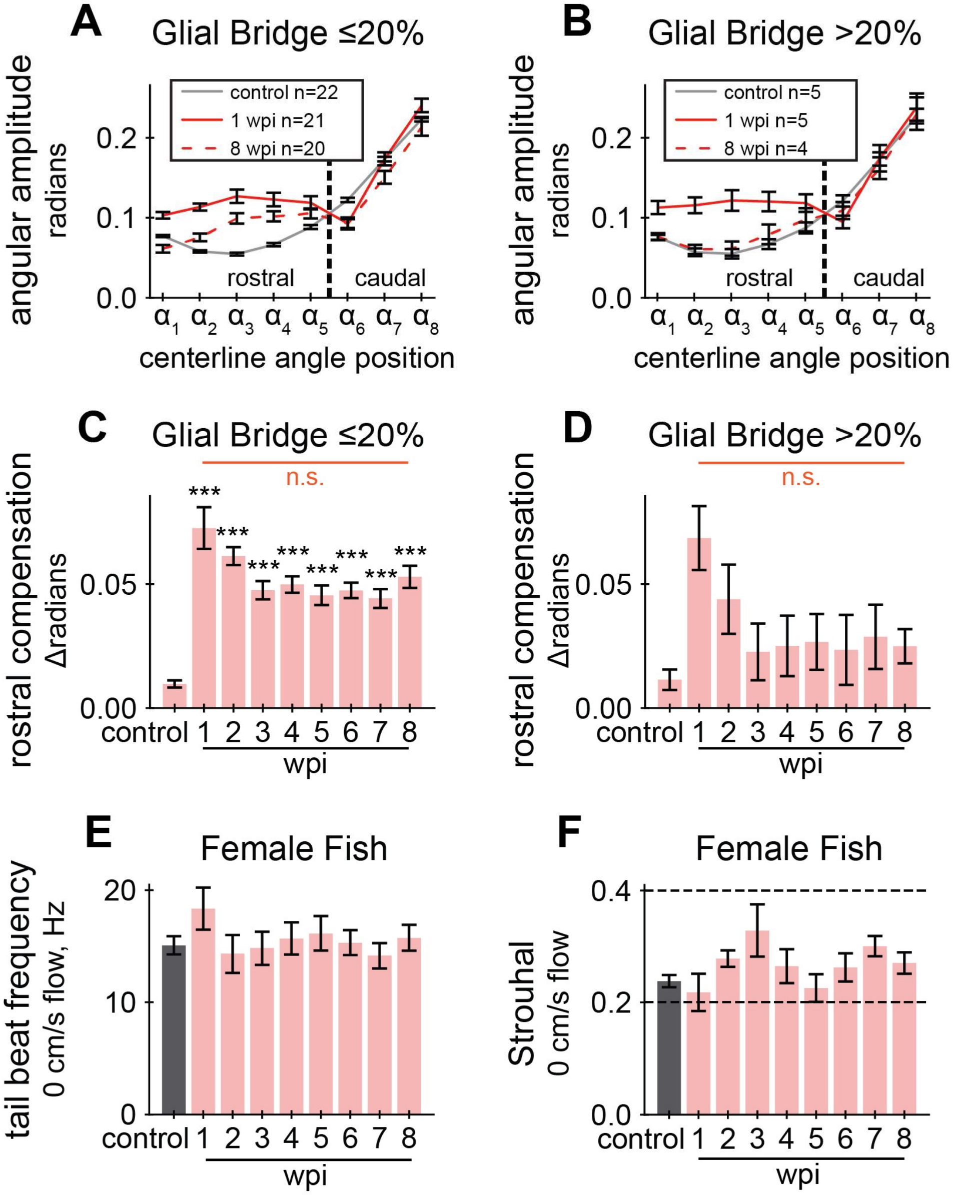

A
Glial Bridge ≤20%
angular amplitude
radians
control n=22
1 wpi n=21
8 wpi n=20
rostral
caudal
centerline angle position
B
Glial Bridge >20%
angular amplitude
radians
control n=5
1 wpi n=5
8 wpi n=4
rostral
caudal
centerline angle position
C
Glial Bridge ≤20%
n.s.
rostral compensation
Δradians
control 1 2 3 4 5 6 7 8
wpi
D
Glial Bridge >20%
n.s.
rostral compensation
Δradians
control 1 2 3 4 5 6 7 8
wpi
E
Female Fish
tail beat frequency
0 cm/s flow, Hz
control 1 2 3 4 5 6 7 8
wpi
F
Female Fish
Strouhal
0 cm/s flow
control 1 2 3 4 5 6 7 8
wpi

Figure S3. Cruise gait quality quantifications for female fish in the tracking experiment. **(A)** Cruise curvature profiles (control, 1wpi, 8wpi) for female fish that regenerated less than 20% of the glial tissue at the lesion site. Each cruise curvature profile represents the mean lateral angular amplitude along the dorsal centerline while cruising. The vertical dotted line separates rostral and caudal positions, demonstrating that acutely injured fish swim with markedly elevated curvature in the rostral portion of their body. **(B)** Cruise curvature profiles (control, 1wpi, and 8wpi) for female fish that regenerated more than 20% of the glial tissue at the lesion site. **(C)** Quantification of rostral compensation in female fish that regenerated less than 20% of the glial tissue at the lesion site. Rostral compensation represents the displacement between cruise profiles. This score is the maximum distance, on the vertical axis, between rostral positions of a cruise profile from the control profile. Angle positions α_1 to α_5 from Panel D were used to define the rostral region. **(D)** Rostral compensation scores for female fish that regenerated more than 20% of the glial tissue at the lesion site. **(E)** Tail beat frequency for female fish in still water (0 cm/s), measured at each assayed week. ANOVA was not significant for tail beat frequencies. **(F)** Strouhal numbers for female fish in still water (0 cm/s), measured at each assayed week. Strouhal number is a unitless value related to vortex shedding mechanics and is defined as tail beat frequency times peak-to-peak amplitude of the tail tip divided by speed of forward motion. ANOVA was not significant for Strouhal numbers. Panels A and C include 22, 22, 21, 20, 20, 20, 20, 19, 20 fish at control, 1, 2, 3, 4, 5, 6, 7, 8 wpi assays, respectively. Panels B and D include 5, 5, 5, 4, 4, 5, 4, 4, 4 fish at control, 1, 2, 3, 4, 5, 6, 7, 8 wpi assays, respectively. Panels E-F include 20, 7, 7, 5, 10, 12, 11, 11, 14 fish at control, 1, 2, 3, 4, 5, 6, 7, 8 wpi assays, respectively. Error bars depict SEM and statistical significance was determined by Brown-Forsythe and Welch's ANOVA tests with Dunnett's T3 multiple comparisons tests. p-value markers in black represent comparisons between each time point post-injury relative to control measurements prior to injury. Red horizontal bars and p-values marked in red show significance between 1 wpi and 8 wpi. ***P<0.001; **P<0.01; *P<0.05; ns, P>0.05.

Figure S4

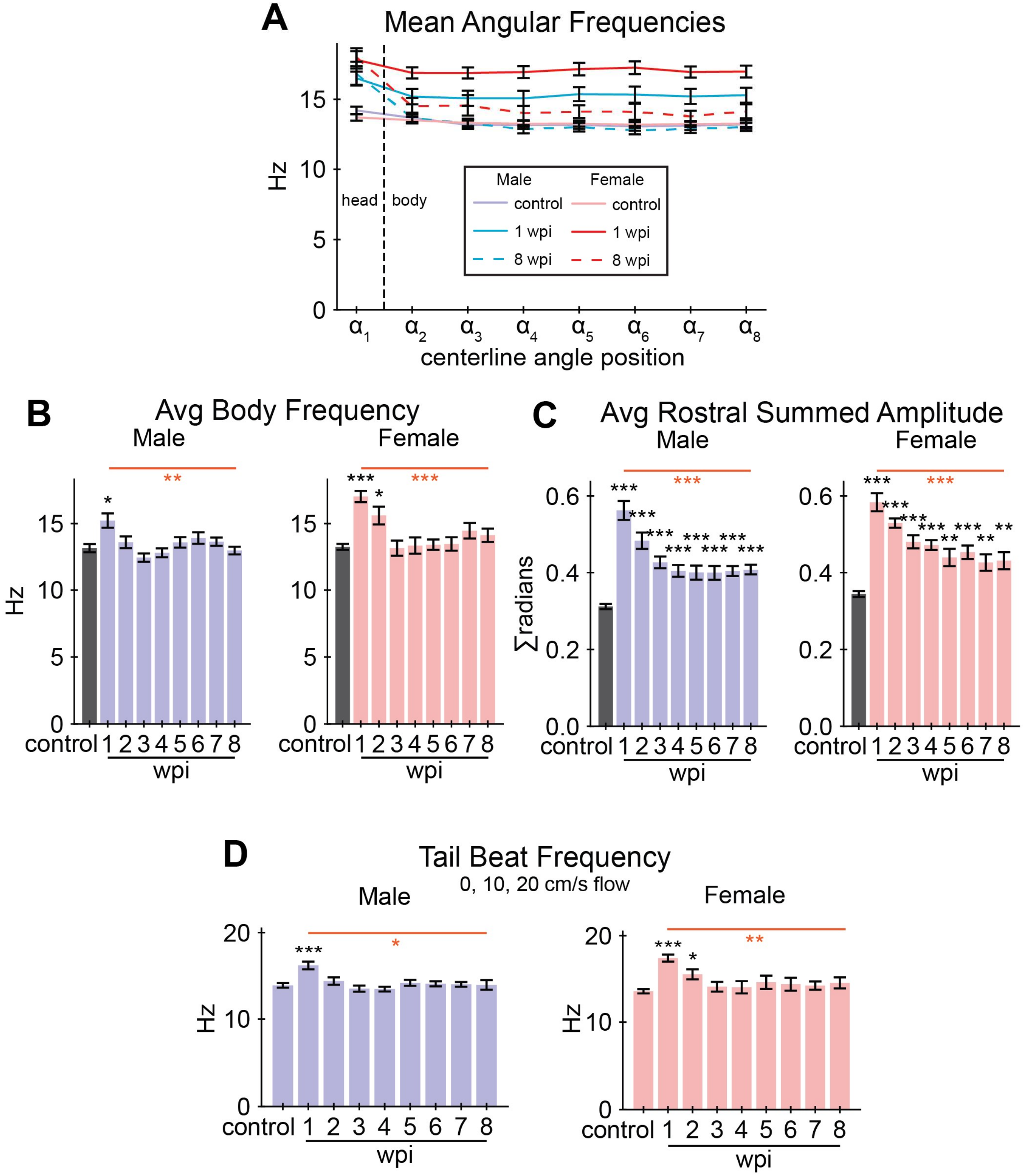
A
Mean Angular Frequencies
Hz
head
body
Male
Female
control
control
1 wpi
1 wpi
8 wpi
8 wpi
centerline angle position
B
Avg Body Frequency
Male
Female
control
wpi
C
Avg Rostral Summed Amplitude
Male
Female
∑radians
control
wpi
D
Tail Beat Frequency
0, 10, 20 cm/s flow
Male
Female
Hz
control
wpi

Figure S4. Cruise waveform features for male and female fish in the tracking experiment. We measured center-to-peak (half of peak-to-peak) curvature amplitude at all angle positions during cruise oscillations. We also measured cruise frequency as the number of complete oscillations at each angle position per second. The body of the fish corresponds to angle positions α_2 to α_8. The rostral region of the fish corresponds to angle positions α_1 to α_5. This figure includes 30, 30, 29, 28, 26, 27, 26, 26, 26 male fish at control, 1, 2, 3, 4, 5, 6, 7, 8 wpi assays, respectively, and includes 30, 28, 26, 24, 24, 25, 24, 23, 24 female fish at control, 1, 2, 3, 4, 5, 6, 7, 8 wpi assays, respectively. **(A)** Mean angle frequencies at each angle position on the centerline (x-axis) measured during cruise episodes, plotted for control, 1 wpi, and 8 wpi. Frequency was stable along the body of the centerline regardless of injury. **(B)** Average angular frequency along body positions for each assay and separated by sex. **(C)** The average sum of angular amplitudes measured at rostral positions along the fish, plotted for each assay and separated by sex. **(D)** Mean tail beat frequency measured throughout each 15-minute swim assay, including all three periods of flow velocity, plotted for each assay and separated by sex. Unlike tail beat frequency measured in the absence of flow (0 cm/s), when we measured tail beat frequency throughout the entire assay the measurement was significantly disrupted by injury, and its recovery plateaued by 3 wpi similar to other gait-related metrics described in the study. Error bars depict SEM and statistical significance was determined by Brown-Forsythe and Welch's ANOVA tests with Dunnett's T3 multiple comparisons tests. p-value markers in black represent comparisons between each time point post-injury relative to control measurements prior to injury. Red horizontal bars and p-values marked in red show significance between 1 wpi and 8 wpi. ***P<0.001; **P<0.01; *P<0.05; ns, P>0.05.

Figure S5

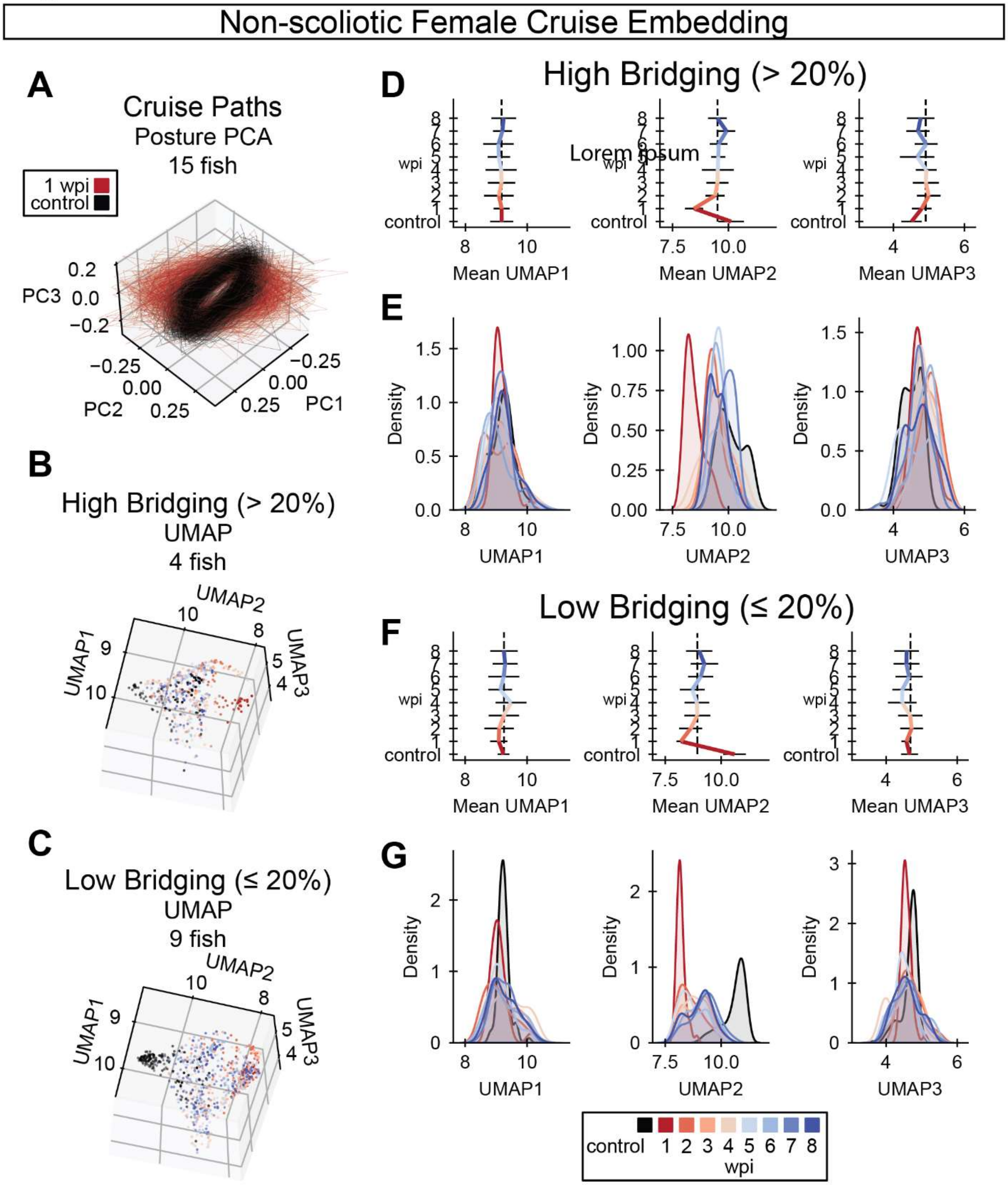
Non-scoliotic Female Cruise Embedding
A
Cruise Paths
Posture PCA
15 fish
1 wpi
control
PC1
PC2
PC3
B
High Bridging (> 20%)
UMAP
4 fish
UMAP1
UMAP2
UMAP3
C
Low Bridging (≤ 20%)
UMAP
9 fish
D
High Bridging (> 20%)
wpi
Lorem ipsum
Mean UMAP1
Mean UMAP2
Mean UMAP3
E
Density
F
Low Bridging (≤ 20%)
G
control 1 2 3 4 5 6 7 8
wpi

Figure S5. UMAP embedding of cruise behaviors during SC regeneration. (A) Cruise poses from control (black) and 1 wpi (red) female zebrafish were decomposed using principal component analysis (PCA). Female fish with scoliosis score < 0.35 at 8 wpi are shown. In this posture space, cruise trajectories from acutely injured fish at 1 wpi are markedly distinct from control fish trajectories. (n=15 fish) **(B-C)** Cruise poses were embedded using UMAP with precomputed dynamic time warping (DTW) distances. Cruise poses were analyzed for fish that regenerated well (glial bridging > 20%) in B and for fish with compromised cellular regeneration (glial bridging < 20%) in C. (Panel B: n=4 fish, panel C: n=9 fish) **(D)** Gait recovery trendlines in UMAP 1, 2, and 3 (left to right) for fish that regenerated well. Colored bars indicate the mean trend of each UMAP component. Error bars are standard deviations. The vertical dotted black lines align with the average UMAP value at 3 wpi. (n=4 fish) **(E)** Gaussian kernel density estimates for the regions of UMAP space occupied by cruises at each week post-injury for fish that regenerated well. The x-axes are matched between Panels D and E for comparison. (n=4 fish) **(F)** Gait recovery trendlines in UMAP 1, 2, and 3 (left to right) for fish with compromised cellular regeneration. Colored bars indicate the mean trend of each UMAP component. Error bars are standard deviations. The vertical dotted black lines align with the average UMAP value at 3 wpi. (n=9 fish) **(G)** Gaussian kernel density estimates for the regions of UMAP space occupied by cruises at each week post-injury for fish with compromised cellular regeneration. The x-axes are matched between Panels F and G for comparison. (n=9 fish)

Figure S6

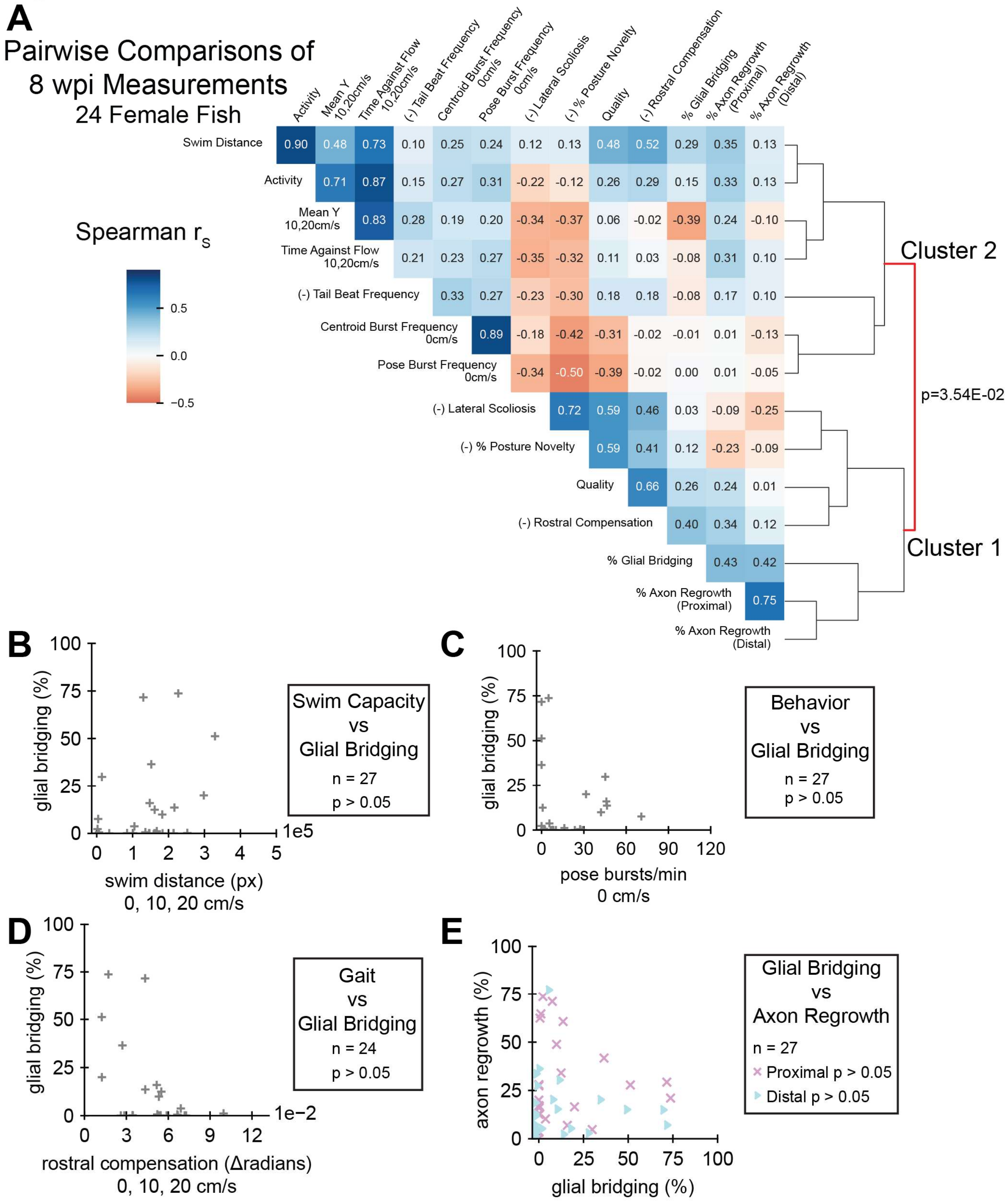
A
Pairwise Comparisons of 8 wpi Measurements
24 Female Fish
Spearman r_s
Swim Distance
Activity
Mean Y 10,20cm/s
Time Against Flow 10,20cm/s
(-) Tail Beat Frequency
Centroid Burst Frequency 0cm/s
Pose Burst Frequency 0cm/s
(-) Lateral Scoliosis
(-) % Posture Novelty
Quality
(-) Rostral Compensation
% Glial Bridging
% Axon Regrowth (Proximal)
% Axon Regrowth (Distal)
Cluster 2
Cluster 1
p=3.54E-02
B
glial bridging (%)
swim distance (px) 0, 10, 20 cm/s
Swim Capacity vs Glial Bridging
n = 27
p > 0.05
C
pose bursts/min 0 cm/s
Behavior vs Glial Bridging
n = 27
p > 0.05
D
rostral compensation (Δradians) 0, 10, 20 cm/s
Gait vs Glial Bridging
n = 24
p > 0.05
E
axon regrowth (%)
glial bridging (%)
Glial Bridging vs Axon Regrowth
n = 27
Proximal p > 0.05
Distal p > 0.05

Figure S6. Pairwise comparisons of spinal cord regeneration metrics at 8 wpi. (A) Heatmap of Spearman's rank correlations (r_s) between the cellular and functional measurements taken. Regeneration metrics include swim capacity, swim quality, structural, and neurological measurements. A dendrogram representing similarities between correlation patterns of measurements is shown. Female fish at 8 wpi were analyzed. Fish that could not be measured in all attributes were omitted. Correlations are reported regardless of p-value. For the sake of meaningful clustering, a measured attribute was multiplied by -1 if its average was increased at 1 wpi compared to controls. These attributes are marked with the prefix "(-)" on the label. (n=24) **(B-D)** One metric each of swim capacity (swim distance), behavior (burst frequency), and gait (rostral compensation) were plotted against glial bridging to demonstrate their relationship, though the correlation p-values were not significant. (Panels B-C: n=27; panel D: n=24) **(E)** Scatter plot showing a non-significant association between the size of regenerated glial tissue and axon regrowth, proximal and distal. The size of regenerated glial tissue plotted against axon regrowth, proximal and distal. r_s were not significant for Panels B-E, likely because most female fish in this experiment exhibited compromised glial regeneration. (n=27)

Figure S7

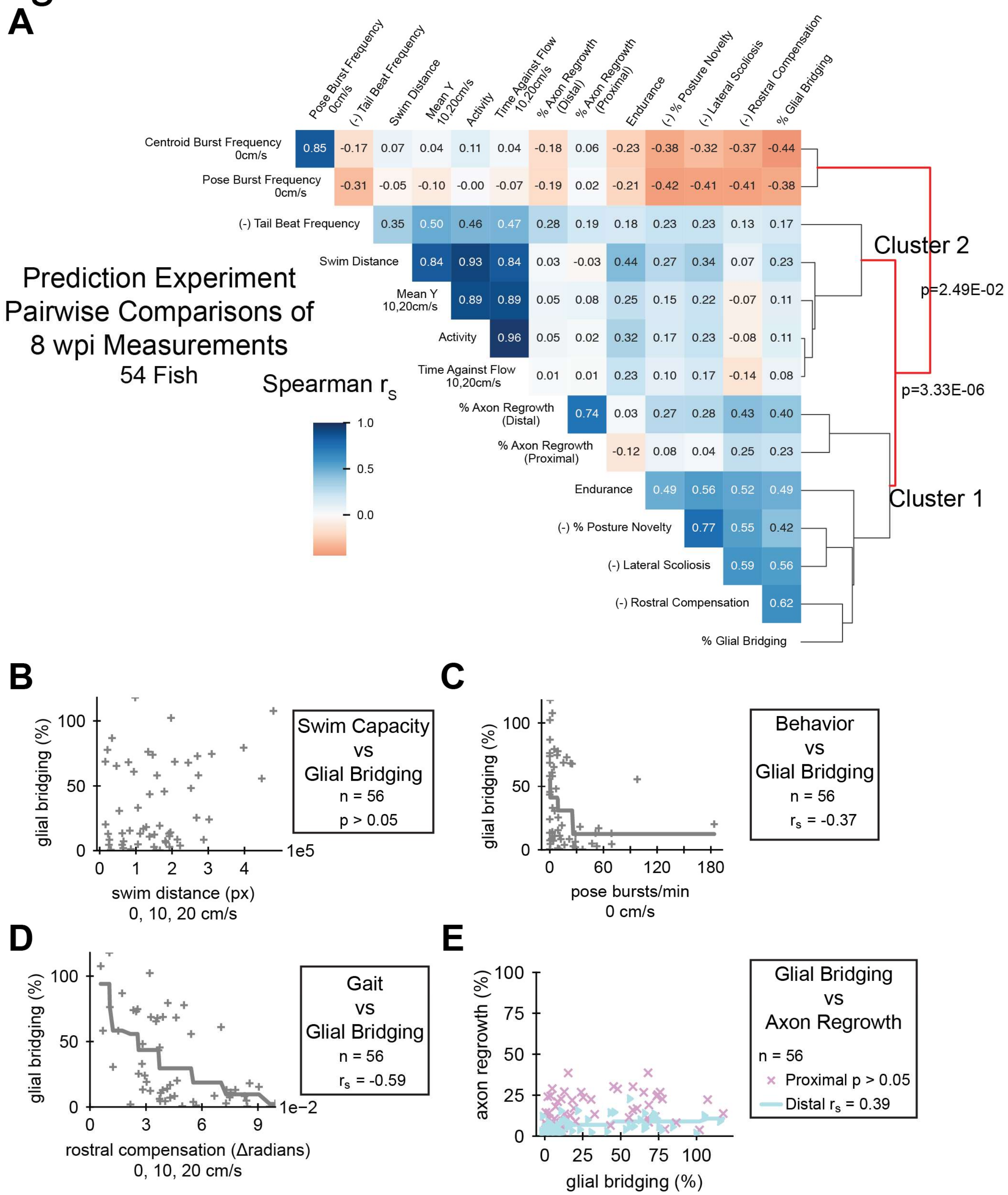

A
Prediction Experiment
Pairwise Comparisons of
8 wpi Measurements
54 Fish
Spearman r_s
Cluster 2
Cluster 1
p=2.49E-02
p=3.33E-06
B
Swim Capacity vs Glial Bridging
n = 56
p > 0.05
glial bridging (%)
swim distance (px)
0, 10, 20 cm/s
C
Behavior vs Glial Bridging
n = 56
r_s = -0.37
pose bursts/min
0 cm/s
D
Gait vs Glial Bridging
n = 56
r_s = -0.59
rostral compensation (Δradians)
0, 10, 20 cm/s
E
Glial Bridging vs Axon Regrowth
n = 56
Proximal p > 0.05
Distal r_s = 0.39
axon regrowth (%)
glial bridging (%)

Figure S7. Prediction experiment: pairwise comparisons of spinal cord regeneration metrics at 8 wpi. (A) Heatmap of Spearman's rank correlations (r_s) between the cellular and functional measurements taken. Regeneration metrics include swim capacity, swim quality, structural, and neurological measurements. A dendrogram representing similarities between correlation patterns of measurements is shown. Male and female fish at 8 wpi were analyzed. Fish that could not be measured in all attributes were omitted. Correlations are reported regardless of p-value. For the sake of meaningful clustering, a measured attribute was multiplied by -1 if its average was increased at 1 wpi compared to controls. These attributes are marked with the prefix "(-)" on the label. (n=54) **(B-D)** One metric each of swim capacity (swim distance), behavior (burst frequency), and gait (rostral compensation) were plotted against glial bridging to demonstrate their correlation. Because the Spearman correlation operates on ranked values, we fit monotonic splines to plots where r_s were significant ($p < 0.05$) to visualize possible associations. (n=56) **(E)** Scatter plot showing a weak association between the size of regenerated glial tissue and axon regrowth, proximal and distal. Axon regrowth was measured lower than expected in this experiment. (n=56)
